# Tunable Capillary Suspensions from Aqueous Two-Phase Systems

**DOI:** 10.1021/acs.langmuir.5c00749

**Published:** 2025-04-28

**Authors:** Leonardo Ruiz-Martínez, Frans Leermakers, Simeon Stoyanov, Jasper van der Gucht

**Affiliations:** † Physical Chemistry and Soft Matter, Wageningen University and Research, Wageningen 6708 WE, The Netherlands; ‡ Food, Chemical, and Biotechnology cluster, Singapore Institute of Technology, 10 Dover Drive, Singapore 138683, Singapore

## Abstract

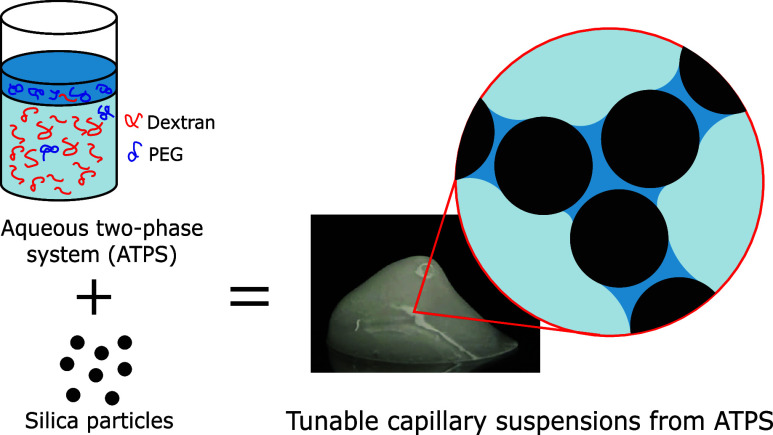

Adding small amounts of a (partially) immiscible fluid to a suspension can create liquid bridges between particles, leading to interconnected networks known as capillary suspensions. This can be used to structure suspensions and adjust their rheological properties. Typically, these suspensions involve water and oil, where the minority liquid phase wets the particles dispersed in the majority phase. Here, we have demonstrated that oil-free capillary suspensions can also be formed in aqueous two-phase systems (ATPS), where a phase separation occurs between two hydrophilic polymers, dextran and polyethylene glycol (PEG). In this system, silica particles form a self-standing gel when a small amount of the PEG-rich phase is added to the dextran-rich phase. Despite the ultralow interfacial tension in ATPS, a significant increase in storage modulus is achievable. Capillary bridges have been visualized using confocal microscopy. By adjusting the amount of the PEG-rich phase (secondary phase), the network strength and yield stress can be finely tuned, enabling a wide range of rheological responses. Due to the absence of oil and the use of hydrophilic, biocompatible polymers, these capillary suspensions have potential applications in biomedical (where living cells can act as particles), pharmaceutical, and food formulations, as well as in home and personal care products.

## Introduction

The rheology of suspensions can be strongly influenced by the addition of a small amount (usually less than 1% volume fraction) of a second immiscible fluid that connects the particles, leading to so-called capillary suspensions. These are structured fluids composed of particles suspended in a continuous liquid phase and linked by capillary bridges made of the secondary liquid.^1,2^ When the amount of secondary liquid is enough to generate a sample-spanning network, the suspension experiences a transition from fluid- to semisolid or gel-like behavior. In fact, the presence of a secondary liquid lowers the percolation threshold of a suspension, with jammed suspensions obtained at solid volume fractions as low as 10%.^3^ Rheologically, the formation of the network leads to the transition from a viscous liquid to a viscoelastic solid with a finite yield stress.^4,5^

The stability and rheological properties of capillary suspensions made from smooth, spherical particles, are expected to depend on several parameters such as the amount of secondary phase, volume fraction of the particles, interfacial tension between the two fluids, particle size (radii), contact angle, number of contacts per particle and material properties.^6−8^ For nonspherical particles, their shape, surface roughness and porosity also play an important role.^9^ For example, the yield stress has been shown to increase with higher interfacial tension and decreasing particle radius.^7^ Similarly, the yield stress of capillary suspensions with pendular bridges is proportional to the cosine of the three-phase contact angle and to the volume fraction of particles.^7^

These relationships demonstrate the richness and versatility of capillary suspensions and the multitude of independent parameters that allow the fine-tuning of the suspension rheology in a very broad range spanning orders of magnitude. Such versatility has triggered a lot of recent research in capillary suspensions for diverse applications, including the use of capillary suspensions as templates for the production of highly porous ceramics and glass filters with narrow pore size distribution,^10−12^ membranes with high electrical conductivity,^13^ and high energy density Li-ion battery electrodes.^14,15^ Other applications comprise the design of conductive pastes for printable electronics^16^ and PDMS-based 3D printing inks which can be printed directly in aqueous medium.^17^

Although the research for these diverse applications has served as an excellent platform to study and understand the new concept of capillary suspensions, most of this research has been focused on the study of nonbiocompatible systems. The inherent lack of biocompatibility is primarily ascribed to the selection of constituent elements, specifically the utilization of liquid mixtures such as synthetic plasticizers (e.g., DINP^18^ and Hexamoll DINCH^19^) and oils as the continuous phase and water as secondary phase. Within the limited reported literature of biocompatible capillary suspensions, notable examples include the formulation of low-calorie cocoa pastes wherein edible oils are incorporated as the secondary phase,^20^ as well as the development of 3D printing inks based on polydimethylsiloxane (PDMS), thereby producing flexible silicone structures.^17^ Another recent study demonstrated the formation of capillary suspensions from aqueous PEG–dextran solutions to structure living cell suspensions,^21^ suggesting potential for broader biomedical applications. Nonetheless, it is important to acknowledge that the inclusion of oils and silicones occasionally raises concerns such as their allergenicity upon contact with biological tissues, especially in the context of biomedical, food and cosmetic applications. Additionally, these constituents face pressing regulations and environmental and sustainability concerns in such industries that lead to a constant search for replacement in consumer products.^22^ Consequently, there exists a compelling impetus to explore more the development of oil-free and water-based alternatives, thereby broadening the potential applications of water-based capillary suspensions.

In this context, we forward an oil-free capillary suspension based on an aqueous two-phase system (ATPS) in which a dextran (polysaccharide) rich phase coexists with a poly(ethylene glycol) PEG rich phase, while both polymers are in a common solvent (water). This is a quasi-two-component system because water may be seen as a spectator, and in this way the ATPS resembles the binary oil–water phases in the classical systems. Therefore, by controlling phase separation so that one coexisting aqueous phase bridges the particles while the other forms the continuous phase, we achieve an oil-free capillary suspension.

Segregative phase separation in ATPS, as in PEG and dextran in water, is driven by repulsive interactions between PEG and dextran, possibly assisted by a difference in affinities for water. As a consequence, phase separation in ATPS is affected by factors such as the concentration and molecular weight (MW) of the polymer and the concentration and composition of salt. For example, long polymers have relatively little mixing entropy, as is clear from Flory–Huggins theory, and hence they tend to segregate at a lower concentration threshold than short polymers. In addition, other factors such as the polydispersity and branching of the polymers may affect the actual phase behavior. Therefore, it is important to understand the phase behavior of the specific system at hand, typically represented through a phase diagram.

The phase diagram serves as a “fingerprint” for ATPS under defined conditions (e.g., temperature, pH, ionic strength). This type of diagram provides a set of concentrations of the components for two-phase formation and their concentration in the top and bottom phases, therefore showing the potential working area to generate two coexisting phases.^23−25^ In a phase diagram with component concentrations on each axis, the binodal curve separates the concentrations at which two phases are formed (above the binodal) from the concentrations at which one phase is observed (below the binodal). Simultaneously, the lines connecting the concentrations of the two coexisting phases are called tie-lines and thus, all pairs of concentrations in such tie line will yield the same top and bottom phase equilibrium compositions.

Several techniques are available to generate phase diagrams, ranging from general methods like cloud-point titration to more specific techniques that exploit component properties, or combinations of these. Cloud-point titration, a widely applicable method, leverages the turbidity that appears as a second phase begins to form in an ATPS. Other methods, such as polarimetry, densimetry, size exclusion chromatography and refractometry, depend on specific properties of the components and are suited to measuring phase boundaries in various system compositions.^26−28^ Extensive data already exists on the phase diagrams and interfacial tension of PEG–dextran mixtures, particularly for certain molecular weights, though these can vary widely across systems. In this work, we use cloud-point titration to determine the phase diagram for our specific polymer batch, complemented by refractive index and interfacial tension measurements to fully characterize our ATPS.

Controlling the phase separation of the PEG–dextran ATPS allows a precise tuning of its properties, such as interfacial tension and volume fraction of each phase. For instance, the interfacial tension has been shown to vary with a scaling exponent of 3/2 as a function of the distance polymer concentration from the critical concentration, as predicted by mean-field theory and confirmed experimentally by Liu et al.^29^ Closer to the critical point, however, they found a slightly higher scaling exponent of 1.67. ATPS interfacial tensions can drop well below 1 mN/m—far lower than typical oil–water values of about 30 mN/m—yet they remain tunable across several orders of magnitude with the described scaling, as demonstrated by Liu et al.^29^ Therefore, polymer concentration (or equivalently, water content) serves as a key parameter for adjusting the interfacial properties in ATPS which is relevant for all types of capillary suspensions.

When solid particles are introduced into a phase-separated PEG–dextran system, like in the formation of capillary suspensions, the two coexisting aqueous phases can exhibit different affinities for the particle surfaces, which is described by the contact angle that these phases form with the particles. As the PEG-dextran phases approach the critical point, the contact angle can shift significantly, transitioning from partial (>0°) to complete wetting (0°).^30^ The contact angle directly influences the type of capillary bridge formed: for contact angles below 90°, pendular bridges are expected between particles, characterizing the ‘pendular state’ of capillary suspensions.^1^ Conversely, contact angles above 90° typically lead to the ‘capillary state.’ In our system, the PEG-rich phase serves as the minority phase, forming bridges among silica particles, which are preferentially wet by the PEG-rich phase rather than the dextran-rich phase. Consequently, our suspensions are anticipated to be in the pendular state, a configuration linked with stronger structures in capillary suspensions due to enhanced attractive forces.^1^

An additional critical parameter for tuning capillary suspensions in general is the volume fraction of each phase. In classical (oil–water) capillary suspensions, one simply adds the desired amount of the immiscible secondary fluid to a primary continuous phase, thereby treating the minority-phase fraction as an external parameter. By contrast, in a PEG–dextran ATPS, the coexisting phases form at equilibrium according to the phase diagram (which can be determined using the lever rule), which depends on factors such as polymer composition, temperature, and ionic strength. One option is to allow the phases to separate in situ, so that the final volume ratio of top to bottom phase is fixed by these equilibrium conditions. Alternatively, and analogous to classical capillary suspensions, the two phases can be physically separated (e.g., by centrifugation or settling) and subsequently recombined with the solid particles in any chosen ratio—thereby making the minority-phase fraction an independently controlled parameter. This approach offers a straightforward means to tune the capillary bridges and thus modulate the rheological properties. Prior studies (on oil–water suspensions) have shown that a minority-phase fraction of about 1–3% often optimizes the network strength in pendular-state capillary suspensions.^1,2,5^ In the present work, we adopt this second strategy—separating and recombining the PEG–dextran phases—to systematically vary the amount of the secondary phase and thereby investigate its effect on the rheological tunability of this fully aqueous system.

This paper aims to present the capacity of producing oil-free capillary suspensions from aqueous two-phase systems with tunable rheological properties. This was achieved by using the aqueous two-phase system formed by the biocompatible polymers PEG and dextran, which was properly characterized by its phase diagram. Using the two aqueous phases, capillary suspensions were prepared and characterized using visual inspection, rheology and confocal microscopy. The rheological tunability of these suspensions was also studied by varying the amount of secondary liquid at a fixed water content.

## Materials and Methods

### Materials

Aqueous two-phase systems were prepared using stock solutions of dextran obtained from *Leuconostoc mesenteroides* (number-average *M*_w_ 150,000 Da) and polyethylene glycol (PEG, number-average *M*_w_ 20,000 Da) acquired from Sigma-Aldrich. The antimicrobial sodium azide was added to stock solutions and both polymers were used without further purification. Capillary suspensions were produced using the aforementioned polymers and silica particles with an average particle size of 1.5 μm (FIBER OPTIC CENTER Inc., USA) and a standard deviation lower than 10% according to the supplier. The silica particles were supplied as a dry powder. These particles are unmodified and highly hydrophilic, as confirmed by the manufacturer’s specifications and by our zeta potential measurements in 1 mM KCl deionized water (approximately −50 mV), which indicate a high density of surface silanol groups and excellent dispersibility in aqueous media. FITC-dyed PEG with an average *M*_w_ 10,000 Da (Biopharma PEG Scientific Inc., USA) was used for confocal imaging of capillary suspensions.

### Preparation of Stock Solutions

Concentrated stock solutions of 30% by weight of both dextran and PEG were prepared. This involved the dispersion of each polymer in deionized water containing 0.02% sodium azide as preservative (ionic strength of the solutions was 0.00308 M). The dispersion process was carried out over an overnight period by using a magnetic stirrer. Continuous stirring on a hot plate up to 80 °C can be used to facilitate the dispersion of dextran. These prepared stock solutions were subsequently stored in a refrigerated environment to prevent UV-induced oxidation and microbial degradation. The stock solutions were brought to room temperature (20 °C) before further use.

### Phase Diagram of PEG–Dextran ATPS

The phase-separating behavior of the PEG-dextran ATPS was characterized by determining its phase diagram with the binodal and the critical point at constant pressure and temperature. In this study, we established the binodal for the dextran and PEG system using cloud-point titration at a controlled temperature of 20 ± 0.5 °C,^26^ while the critical point was determined by assessing the volume fraction of each phase along various dilution lines.

In the titration process, a one polymer solution at specific concentrations was prepared by diluting stock solutions in 14 mL vials. The complementary polymer stock solution was added drop by drop, with constant stirring provided by a magnetic bar. Titration proceeded until turbidity indicated the formation of a second phase, marking the two-phase region. This process was repeated in both directions: from a one-phase to a two-phase region (turbid) and back (clear), with all masses of polymer and water precisely measured using a high-precision balance.

To determine the critical point, solutions with equal polymer weight ratios and varying water content were prepared and equilibrated until complete phase separation. This was achieved by waiting for several hours, and then centrifuging at 1500*g* for 15 min. This process was repeated for a series of different polymer weight ratios. The critical point was then estimated by analyzing the evolution of phase volumes as the binodal was approached within the two-phase region. At the critical point, the volume of both phases become equal phase.^29^

### Preparation and Characterization of PEG–Dextran ATPS for Capillary Suspensions

First, the stock solutions were thoroughly shaken to prevent the formation of density gradients. Subsequently, a 15 mL Falcon tube was positioned on a precision weighing balance, and the stock solutions were added according to the desired polymer concentration and the total amount of mixture needed (usually 10–15 g). The quantification of the stock solutions was carried out by weight due to the high viscosity of the highly concentrated solutions. These solutions were introduced in descending order of their densities, stratified in layers to facilitate the potential removal of portions in case of weighing errors. The mixture was agitated for at least 1 min using a vortex mixer until a uniformly cloudy appearance was achieved.

**Figure 1:**
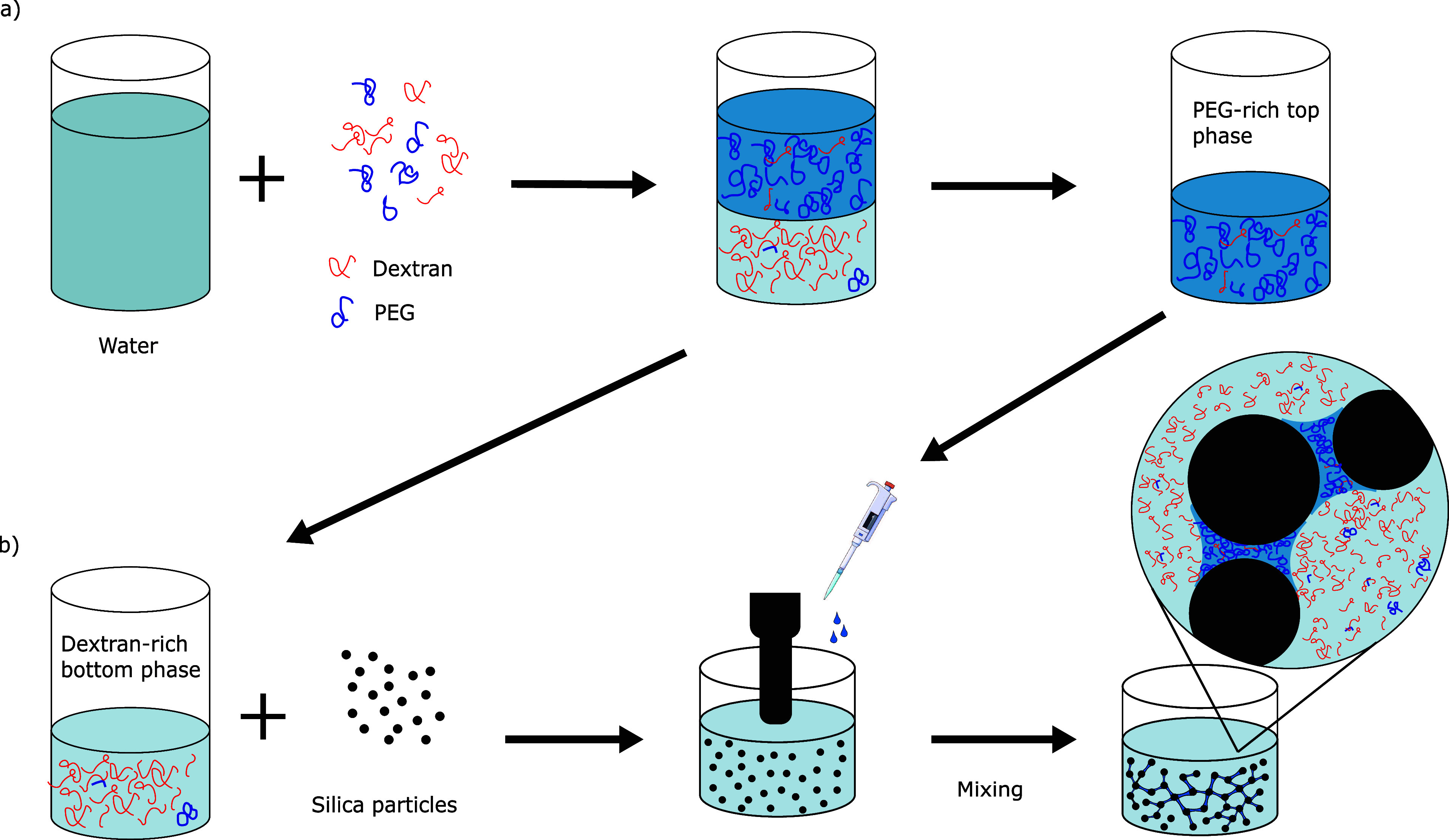
Schematic drawing of (a) the preparation process of the PEG–dextran aqueous two-phase system, and (b) the manufacturing process of capillary suspensions formed by silica particles (ϕ_solid_ = 25%) suspended in dextran-rich phase and containing small amounts of PEG-rich phase as secondary phase.

For the subsequent utilization of the prepared aqueous two-phase system (ATPS) in the production of capillary suspensions, the mixture underwent phase separation through centrifugation (utilizing a Multifuge X1R, Thermo Fisher Scientific) at 1500g for a duration of 15 min. The PEG-rich and dextran-rich phases were extracted and stored separately in designated containers. A schematic representation of the ATPS preparation process is illustrated in Figure 1a. Due to practical issues such as the volume and viscosity of each phase, the ATPS used in the preparation of the aqueous capillary suspensions in this work were obtained using a total polymer concentration (PEG and dextran) of 30% and a polymer weight ratio (*w*_d_/*w*_p_) of 2, unless otherwise mentioned.

Due to practical considerations such as differences in volume and viscosity, the ATPS used in this work was prepared at a total polymer concentration of 30 wt % (combined dextran and PEG) and a polymer weight ratio (*w*_d_/*w*_p_) of 2, unless otherwise specified. Based on refractometric measurements, mass balance, and the phase diagram, we estimate that the dextran-rich phase exhibits a total polymer concentration of approximately 37.4 wt %, consisting almost entirely of dextran (with PEG present only in trace amounts on the order of 0.02 wt %). Conversely, nearly all of the PEG (originally 10 wt % of the starting mixture) partitions into the PEG-rich phase, yielding a calculated total polymer concentration of about 21.5 wt % that is essentially composed of PEG, with the dextran content falling below 0.01 wt %.

Lastly, a SVT 15 spinning drop tensiometer (DataPhysics) was used to measure the interfacial tension between the coexisting PEG-rich and dextran-rich phases that corresponded to the tie lines used to prepare the capillary suspensions. The setup consisted of a transparent glass capillary with a 3.5 mm diameter filled with the dextran-rich phase solution into which a 1 μL droplet of the PEG-rich phase solution was injected. The capillary was rotated at a certain speed ω between 1000 and 15,000 rpm, producing the elongation of the PEG-rich phase droplet along the axis of rotation. The Vonnegut equation was used to determine the interfacial tension between the two phases when the droplet length was greater than four times its equatorial diameter.^31^

### Preparation of Capillary Suspensions Using PEG–Dextran ATPS

Capillary suspensions were prepared in 5 mL samples, with 25% of the sample volume being composed of dry silica particles. The remaining volume was allocated to the liquid phases, the precise quantity of each phase depended on the bulk/secondary phase proportion. The preparation of capillary suspensions involved a stepwise procedure.

Initially, the dextran-rich phase was mixed with silica particles for a duration of 2 min using an Ultra-Turrax T10 basic (IKA) operating at 14,000 rpm. Subsequently, 0–5% of the PEG-rich phase (based on total liquid phase volume) was introduced as the secondary phase and agitated for 1 min at the same rotational speed. A schematic representation of this process is depicted in Figure 1b for visual reference.

It is noteworthy that alternative methods for producing capillary suspensions exist. One such approach involves first preparing the ATPS and separating the dextran-rich phase from the PEG-rich phase. These phases are then mixed again in the desired volume ratio of bulk to secondary phase, where the dextran-rich phase serves as the bulk phase and the PEG-rich phase as the secondary phase. This mixture is stirred thoroughly to reduce the size of the PEG-rich domains, ensuring that the PEG-rich phase can effectively form capillary bridges between particles upon their addition, resulting in capillary suspensions.

Yet another method for manufacturing capillary suspensions involves producing the ATPS phases concurrently during the capillary suspension preparation, eliminating the need for preformed ATPS solutions. For this method, the exact concentration of polymers producing two phases and their corresponding volumes need to be known. In this work, we have chosen to separately produce the ATPS since in this way the formation of two phases is checked before proceeding to the formation of capillary suspensions, thus small errors in the manufacturing are more tolerable and it allows to control interdependently the volume fraction of the dispersed phase, similarly to what is done in the case of conventional capillary suspensions.

### Characterization of Capillary Suspensions and Liquid Phases

The liquid to solid transition behavior of capillary suspensions was evaluated using rheology, and their microstructure was elucidated using confocal imaging. Rheological measurements were performed in a stress-controlled rheometer (Physica MCR 501, Anton Paar, Austria) using a plate–plate geometry with a 25 mm plate diameter and a gap of 1 mm. Both bottom and top plates were covered with sandpaper 400 grit to avoid slip.^22^ Oscillatory stress-sweep measurements were performed from 0.01 to 100 Pa at a constant angular frequency of 1 rad/s. Frequency sweeps from 10 to 0.1 rad/s were performed at a constant oscillating shear stress of 1 Pa, a value in which all samples fell within the linear viscoelastic region. All samples were presheared in an oscillatory manner with stresses of 1000 to 10 Pa at 10 rad/s for 1 min to ensure breaking of the internal structure. Afterward, the samples were left to recover for 15 min before running each measurement at 20 °C. All measurements were performed on the same day of preparation.

Complementarily, the microstructure of capillary suspensions was observed using a confocal microscope to properly visualize individual layers inside the sample. For this technique, particles were labeled with rhodamine B using a modified Stober synthesis as described by Bossler.^32,33^ The secondary phase was labeled by replacing 0.15 wt %of PEG with FITC-PEG in the samples. The confocal images were taken with a Nikon C2 confocal microscope (Nikon, Japan). The particle dye was excited using the 561 nm laser and detected in the wavelength range 650–1000 nm while the secondary phase dye was excited at 488 nm and detected in the wavelength range 576–613 nm to avoid interference between signals.^34^

## Results and Discussion

### Dextran–PEG Aqueous Two-Phase System (ATPS)

**Figure 2:**
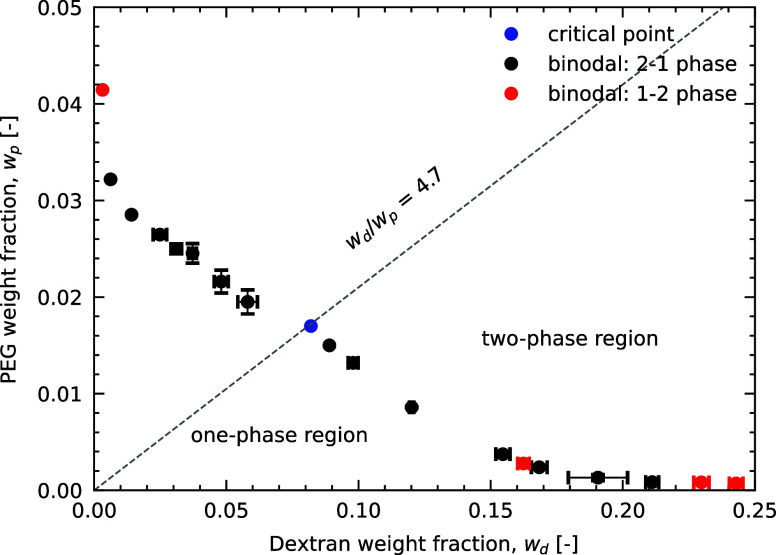
Phase diagram for aqueous two-phase system of PEG and dextran. The points represent the binodal and jump-like transition from the one-phase to the two-phase region. Black points were obtained moving from the two- to the one-phase region while red points were in the opposite direction. The dashed line corresponds to the dilution line with the ratio *w*_d_/*w*_p_ = 4.7 where the critical point (blue) was found.

Success in the manufacturing of capillary suspensions with two aqueous phases depends on the phase-separating ability of the aqueous PEG–dextran system. Therefore, it was important to ensure that two phases were effectively generated and separated before using them for the preparation of capillary suspensions. This was achieved by first determining the concentrations at which the mixture dextran–PEG generates two phases and then, preparing it at those concentrations. The phase-separating concentrations of PEG-dextran were mapped in a phase diagram by using the cloud-point titration technique at 20 °C and atmospheric pressure. This titration was performed from the one-phase region to the two-phase region and vice versa, yielding similar results in both cases. The results of this titration are presented in Figure 2. Example images of the clear one-phase solution and the cloudy two-phase solution, taken during the cloud-point titration, are provided in the Supporting Information (Figures S1 and S2).

This phase diagram depicts the phase-separating behavior of an aqueous solution of dextran and PEG in terms of their weight percentage, denoted by *w*_d_ and *w*_p_, respectively. The diagram consists of three main parts: the binodal curve, the dilution line, and the critical point. The binodal curve (points) corresponds to the boundary of the two-phase coexistence region. Compositions at and below the binodal generate one homogeneous phase while compositions above the binodal generate two coexisting phases: one rich in PEG, and one rich in dextran. The dilution line in the phase diagram corresponds to the lines containing varying water content at a fixed polymer weight ratio as shown in the gray dashed line for the weight ratio dextran/PEG equal to 4.67, which was found to cross the critical point of the PEG–dextran ATPS. At the critical point, the difference in composition and density between the two coexisting phases becomes negligible and therefore the volume of the two phases is equal.^28^ By following this principle, we were able to estimate the critical point around the concentrations of 8.45 wt % dextran and 1.8 wt % PEG, as shown on the graph by the blue point. The data of phase volume fractions for the determination of the critical point is shown in the Supporting Information.

A particular characteristic of aqueous two-phase systems is the low interfacial tension between the coexisting phases, which can vary along dilution lines and is an important parameter in determining the strength of capillary suspensions. For the ATPS used in this work (30% total polymer concentration and *w*_d_/*w*_p_ = 2), we measured an interfacial tension of 542 μN/m, which is approximately 2 orders of magnitude lower than the typical values for oil–water systems (30 mN/m) used in classical capillary suspensions. This low value reflects the aqueous nature of both phases and underscores the marked difference between ATPS and oil–water systems.

In the literature, interfacial tension in polymer-based ATPS is known to follow a power-law relationship with polymer concentration, particularly when plotted as a function of *w*_p_ – *w*_p_^(cr)^, where *w*_p_^(cr)^ represents the critical polymer concentration. Liu et al.^29^ reported that the power-law exponent is consistent with mean-field theory predictions (3/2) except very close to the critical point, where the slope slightly increases to 1.67. This relationship highlights the ability to tune the interfacial tension of ATPS by adjusting the water content (or equivalently, the polymer concentration), offering a simple yet powerful way to control the capillary force between particles and thus, the capillary suspension properties.

### Formation and Imaging of Aqueous Capillary Suspensions Using PEG–Dextran ATPS

Once the aqueous two-phase system (ATPS) PEG–dextran was characterized, it could be used to manufacture capillary suspensions. For the manufacturing of capillary suspensions, the dextran-rich phase was chosen as the bulk phase and the PEG-rich phase as the secondary phase. This choice was based on the observation that the silica particles preferentially partitioned into the PEG-rich phase, as concluded from a simple wetting experiment. In this experiment, a small amount of silica particles was dispersed in a dextran-PEG ATPS and left to rest overnight. Consequently, the two phases separated, and the silica particles moved to the top PEG-rich phase, as evidenced by the highly reflective white phase observed at the top. This behavior indicates a strong preference of silica particles for the PEG-rich phase over the dextran-rich phase, even though the particles are much denser than either liquid phase. Additionally, no particles were observed at the interface between the PEG-rich and dextran-rich phases, suggesting that the contact angle is close to 0°. It should be noted, however, that the contact angle may depend on the water concentration in the system.

**Figure 3:**
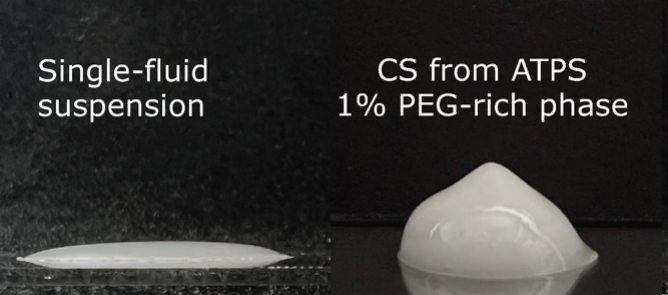
Transition from liquid to gel-like behavior of aqueous suspensions upon introduction of a secondary aqueous phase formed via ATPS. The image depicts samples containing ϕ = 25% silica particles suspended in 40% dextran aqueous solution (left) and in a dextran-rich phase and 1% PEG-rich phase as secondary phase (right), both pictures were taken right after preparation.

Capillary suspensions were obtained using the ATPS with a total polymer concentration of 30% and a polymer weight ratio (*w*_d_/*w*_p_) of 2. Once the ATPS was formed, the two phases were harvested to be used separately and have more control over the manufacturing process. Capillary suspensions were then prepared by first mixing silica particles and the dextran-rich phase (liquid bulk phase), and consequently adding a varying amount between 0 and 5% of PEG-rich phase. The volume fraction occupied by the particles was kept constant at 25%, while the rest of the volume was split between the bulk phase and the secondary phase, where the latter received the minority. Figure 3 shows a picture of the paste formed by a capillary suspension using two aqueous phases. This figure clearly shows the behavior transition from a 25% silica suspension in a 40% dextran solution and the capillary suspension containing 1% of the secondary PEG-rich phase. It is important to note that the reference silica suspension does not contain any PEG or PEG-rich phase added to it. The effect of the presence of PEG in the secondary phase is obvious and directly impacts the behavior of the whole system. In this case it is possible to observe that the presence of only 1% of the secondary phase generates the change in behavior toward a more paste like behavior having good shape retention indicating the presence of yield stress sufficient to counteract the stratigraphic and capillarity pressures which are trying to flatten the paste blob shape. Indeed, the dominance of capillary forces over gravitational effects can be quantitatively confirmed by calculating the Bond (Eötvös) number. Given our experimental parameters—a particle diameter of 1.5 μm, an interfacial tension of 0.542 mN/m, and a density difference of 1400 kg/m^3^—the calculated Bond number is approximately 5.7 × 10^–5^, confirming that capillary forces strongly outweigh gravitational forces in stabilizing the observed particle network.

**Figure 4:**
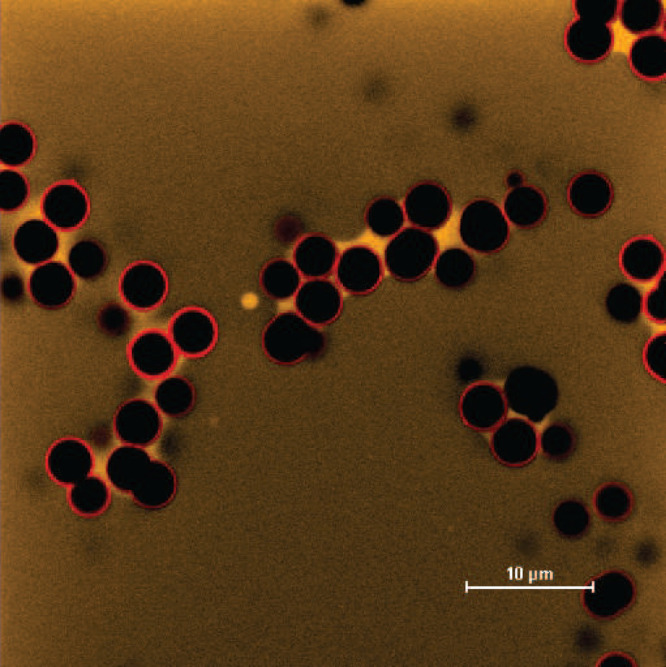
Confocal microscopy images illustrating the formation of individual PEG-rich capillary bridges between silica particles in a diluted suspension. Particles are shown in red while capillary bridges (PEG-rich phase) in yellow.

A fundamental aspect of capillary suspensions lies in the formation of capillary bridges among the particles. Therefore, a natural means to validate the presence of these bridges is through their direct observation using microscopic techniques. In the samples previously discussed, these bridges consisted of the PEG-rich phase, which can be visualized under a confocal microscope using predyed PEG (FITC-dyed PEG). Although the refractive index mismatch between the silica particles and the aqueous phases makes confocal microscopy unsuitable for visualizing dense, sample-spanning networks, we successfully demonstrated the formation of individual capillary bridges by preparing intentionally diluted suspensions. An example confocal image obtained from such a diluted suspension is shown in Figure 4. These bridges appear as areas of high yellow intensity due to the high concentration of PEG within them, while the bulk dextran-rich phase exhibits a lighter yellow hue owing to its limited PEG content. The observed morphology corresponds to a pendular/funicular network, characterized by internal contact angles lower than 90° and small cluster of more than 2 particles.^3^ This phenomenon arises from the superior wetting properties of PEG on silica particles. When these particles are suspended in a a dextran-rich phase saturated with PEG, an amount of the PEG-rich phase is adsorbed onto the silica particles. When two such particles come into proximity, their wetting layers intermingle, giving rise to the formation of the capillary bridge.^35^ It is worth noting that at the nanometer scale a thin layer of the adsorbed PEG-rich phase should exist which remains beyond the resolution capabilities of the microscope used.

### Rheology of Aqueous Capillary Suspensions

The presence of capillary bridges in the aqueous capillary suspensions, as visualized in the microscopic images presented in the previous section (Figure 4), directly influences their rheological properties. These bridges form sample-spanning particle networks that govern the suspension’s mechanical behavior. The capillary forces that drive the formation of these bridges (*F*_c_) are proportional to the interfacial tension (γ) and depend on the particle size (*R*) and the geometry of the bridge, as expressed by *F*_c_ ∝ γ*R* cos θ, where θ is the contact angle.^1^ The yield stress (τ_y_) and modulus (*G*′) of a suspension scale with the strength of these capillary forces and the connectivity of the particle network. As shown by Danov et al.,^7^ the yield stress is proportional to the capillary force and the density of capillary bridges per unit area. In classical capillary suspensions, where immiscible oil–water systems exhibit interfacial tensions of 30–55 mN m^–1^, these strong capillary forces result in high modulus and yield stress values.^18,36^

**Figure 5:**
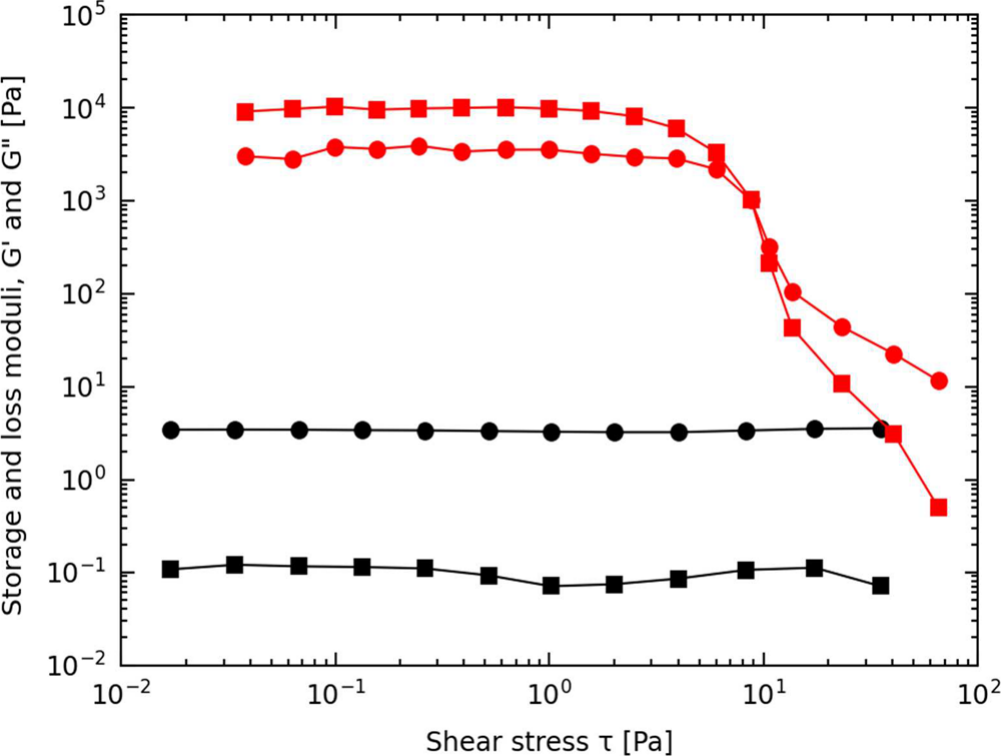
Rheological transition of aqueous suspensions upon the addition of a secondary aqueous phase. Storage (squares-) and loss (circles) moduli vs shear stress at 1 rad/s for capillary suspension with 1% PEG-rich phase added (red) and a corresponding single-fluid suspension containing 40 wt % dextran solution (black).

Figure 5 compares the rheological behavior of a single-fluid suspension, containing 40 wt % dextran, to an aqueous capillary suspension with the same total polymer concentration but with 1% v/v PEG-rich phase added. The single-fluid suspension exhibits a predominantly viscous response, as indicated by the loss modulus (*G*″) exceeding the storage modulus (*G*′) across the entire stress range. Furthermore, *G*″ remains nearly constant, forming a horizontal line that suggests no yield stress and no percolated particle network.

In contrast, the aqueous capillary suspension demonstrates gel-like behavior. The storage modulus (*G′*) exceeds the loss modulus (*G″*) in the linear viscoelastic region, and a flow point at approximately 8 Pa is observed at the crossover point of *G′* and *G″*. Beyond this point, the suspension exhibits shear-thinning behavior, characterized by a decrease in both moduli with increasing stress. Notably, the storage modulus (*G′*) increases by 5 orders of magnitude compared to the single-fluid suspension, underscoring the dramatic impact of capillary bridge formation. These results highlight the role of the PEG-rich phase in generating capillary bridges, which percolate to form a sample-spanning network and induce gel-like properties.

The system is also characterized by the induction of a dynamic yield stress, which can be estimated as the point where the linear elastic regime ends (significant initial drop in storage modulus). The aqueous capillary suspension shown here has a yield stress around 2 Pa. This value demonstrates that a soft gel is obtained compared to the classical capillary suspensions that often present yield stresses 2–3 orders of magnitude higher, mainly because of their higher interfacial tension of tens of mN/m, like in the case of typical silicone oil–water systems (40–55 mN/m).^18,36^ Compared to these classical systems, the softer gels in ATPS-based suspensions reflect the significantly lower capillary forces, yet still demonstrate the potential to induce gel-like behavior.

### Effect of the Amount of PEG-Rich Phase Added

**Figure 6:**
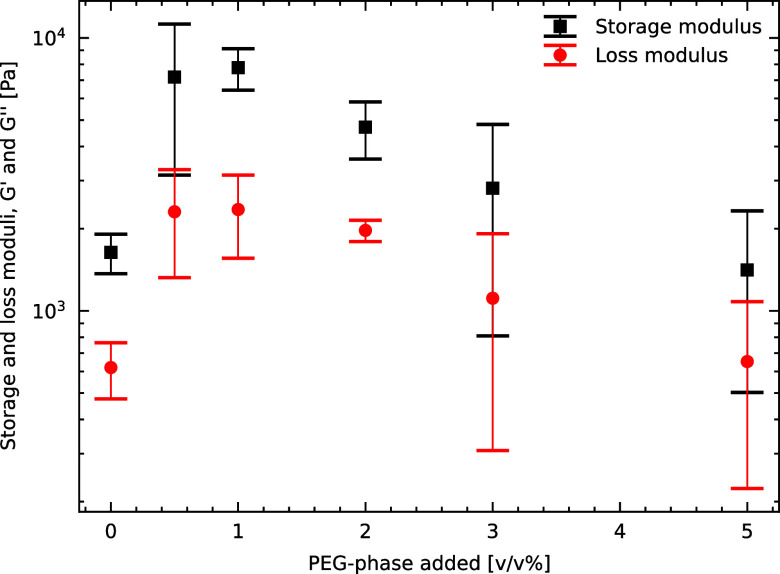
Shear storage and loss moduli at 1 rad/s and 1 Pa of capillary suspensions formed by adding 0–5% of extra PEG-rich phase as secondary phase.

The rheological behavior of capillary suspensions is strongly influenced by the fraction of secondary phase, since it governs the extent of particle bridging. In this study, we systematically varied the amount of PEG-rich phase (the secondary phase) and studied the rheological response of such samples. Figure 6 illustrates the impact of this variation on the storage modulus (*G′*) and loss modulus (*G*″).

The first feature this plot reveals is that capillary suspensions can form even when no additional PEG-rich phase is introduced (0% PEG-rich phase added). This phenomenon arises from the presence of a small amount of PEG dissolved in the dextran-rich phase, which enables PEG to be adsorbed on the particle surface and form capillary bridges between nearby particles. Quantitative estimates indicate that even the low concentration of PEG in the 0% added PEG-rich phase sample (approximately 0.02 wt %, corresponding to about 0.87 mg of PEG) is entirely adsorbed onto the silica surfaces, creating adsorbed PEG layers that promote the formation of capillary bridges. Based on literature values of approximately 0.5 mg PEG adsorbed per m^2^ of silica,^37^ roughly 2.5 mg of PEG would be required to fully saturate all particle surfaces in our samples. Thus, the available PEG in the 0% added sample is insufficient to cover all particle interfaces completely, potentially resulting in fewer and weaker capillary bridges than in samples with higher PEG content. It is important to distinguish this case from the single-fluid suspension described earlier, which lacks any PEG and thus shows no capillary bridge formation or gel-like behavior.

Further examination of the plot shows that the addition of 1% PEG-rich phase leads to the maximum storage modulus (*G′*), indicating the strongest gel structure. In this case, the total PEG mass (approximately 8.5 mg) is sufficient to fully cover the particle interfaces and replenish PEG in the dextran-rich phase, thereby maximizing the network’s capillary forces and strengthening the gel structure. This enhanced strength results from the interplay between several factors, including the number and size of capillary bridges formed and the configuration of the particle network. While the relative contributions of these factors cannot be quantified with the available data, the observed peak behavior is consistent with reports on classical capillary suspensions, such as those by Koos et al.^36^ and Velankar.^3^ This consistency across different systems highlights the robustness of this phenomenon.

As the proportion of PEG-rich phase increases beyond 1%, the gel strength decreases significantly. This decrease occurs because the excessive addition of PEG-rich phase leads to the formation of particle-filled droplets of the secondary phase. These droplets disrupt the connectivity between particles, weakening the gel structure. Similar behavior has been reported in classical capillary suspensions, where excessive secondary phase addition results in a loss of network integrity.^38^ This observation underscores the delicate balance required between the constituents of capillary suspensions and highlights the critical role of the amount of secondary phase in determining their mechanical properties.

### Frequency Dependency of the Shear Moduli

**Figure 7:**
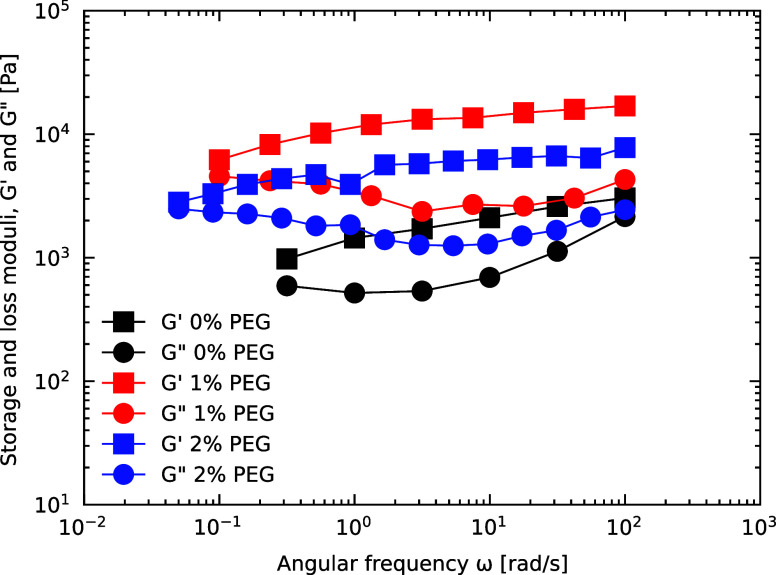
Shear moduli dependency on frequency of capillary suspensions containing 0, 1*,* and 2% of extra secondary added phase. Measurements were done at constant stress of 0.1 Pa.

The frequency dependency of the storage modulus (*G′*) and loss modulus (*G″*) provides insights into the mechanical response of aqueous capillary suspensions at different time scales. Figure 7 shows the moduli, measured on the same day of preparation, as functions of angular frequency (ω) for three representative samples with 0, 1, and 2% PEG-rich phase added. These samples were chosen to demonstrate the characteristic behavior observed across all tested suspensions while simplifying the visualization.

At low frequencies, all samples show a tendency toward a crossover point where *G″* exceeds *G′*. This crossover is not explicitly seen within the frequency range tested but can be inferred from the curves’ behavior. The low-frequency crossover is indicative of a Maxwell-like response, where the loss modulus dominates at frequencies below the inverse of system’s characteristic relaxation time (τ). In capillary suspensions, the relaxation time may reflect the time scale of particle reorganizations within the capillary gel, which are facilitated by the relatively low interfacial tension.

For the 0% PEG-rich phase sample, the weakly aggregated network formed by the PEG-saturated dextran-rich phase relaxes more quickly, leading to a crossover at a higher frequency. In contrast, the 1 and 2% PEG-rich phase samples exhibit slower relaxation dynamics due to stronger and more numerous capillary bridges, causing the crossover to shift toward lower frequencies. This trend highlights how increasing PEG content stabilizes the capillary network, delaying the onset of viscous behavior. Such frequency-dependent behavior aligns with the predictions of the Maxwell model, where the relaxation time (τ = η/*G′*) increases with network strength and particle connectivity.

At high frequencies, the storage modulus (*G′*) dominates for the 1 and 2% PEG-rich phase samples, reflecting the elastic nature of the capillary network at short time scales. For the 0% PEG-rich phase sample, *G″* approaches *G′*, suggesting a weaker network that cannot withstand fast deformations as effectively. The upturn in *G″* observed for all samples at high frequencies can be attributed to the viscous contribution of the bulk dextran solution. For polymer solutions like dextran, *G″* increases linearly with frequency as *G″*∼ ωη, where η is the viscosity of the polymer solution. Using viscosity data for dextran solutions from Heinze et al.,^39^ the viscosity of a 40 wt % dextran solution is approximately 1.5 Pa·s. Based on this value, the estimated *G″* at ω = 100 rad/s is *G″* ≈ ωη = 100 rad/s × 1.5 Pa·s = 150 Pa. This estimation aligns well with the experimental *G″* values for the 0% PEG-rich phase sample, suggesting that the high-frequency behavior is dominated by the dextran matrix’s viscous dissipation. For the 1 and 2% samples, *G″* is higher than expected from the dextran contribution alone, indicating additional energy dissipation mechanisms. These may include viscous dissipation within the capillary bridges or microstructural constraints imposed by the denser particle network.

In conclusion, the frequency-dependent behavior of aqueous capillary suspensions reflects the interplay between capillary bridge dynamics, particle network reorganization, and bulk polymer contributions. At low frequencies, the Maxwell-like relaxation of the network governs the moduli, while at high frequencies, *G″* is dominated by the viscous dissipation from the dextran matrix and capillary bridges, and *G″* is determined by the particle network structure.

### Broader Implications and Future Directions

This study demonstrates the ability of aqueous capillary suspensions to exhibit tunable rheological properties, opening a range of possibilities for both fundamental research and practical applications. While the present work focuses on the amount of PEG-rich phase as a key tunable parameter, several additional factors could influence the mechanical properties of these systems.

#### Potential for Further Tuning

The polymer ratio, concentration, and overall water content directly influence the phase behavior of the PEG-dextran aqueous two-phase system (ATPS). These factors impact the interfacial tension between phases, the wettability of particles, and the resulting network strength. By varying these parameters, it would be possible to achieve a wider range of viscoelastic properties. For instance, reducing the water content in the system might enhance the capillary bridge strength by increasing the interfacial tension, potentially leading to higher storage modulus (*G′*) and yield stress values. Additionally, adjusting the polymer ratio could further influence the interfacial properties and particle interactions.

Another intriguing aspect of water-continuous capillary suspensions is the highly dynamic nature of the liquid–liquid interfaces. The exchangeability of the PEG and dextran phases may lead to aging effects, where the capillary bridges reorganize over time, potentially affecting the suspension’s stability and rheological response. Studying these aging phenomena could provide insights into the long-term behavior of aqueous capillary suspensions, particularly for applications where stability is critical.

#### Broader Implications and Challenges

Beyond the specific characteristics of PEG-dextran ATPS, other liquid–liquid phase separation mechanisms could be explored to design capillary suspensions tailored for specific needs. For example, using complex coacervation of polyelectrolytes or thermally induced phase separation could expand the range of phase-separated systems available for structuring aqueous suspensions. These alternative systems might introduce additional tunable parameters, such as charge density, ionic strength, or temperature, further diversifying the potential applications.

A key challenge for future research is understanding the extent to which characteristics of traditional capillary suspensions, such as particle loading, size, and shape, translate to water-continuous systems. While these factors have been extensively studied in oil–water systems, their interactions with liquid–liquid phase separation in ATPS require further investigation. For instance, smaller particles might lead to thinner capillary bridges, enhancing sensitivity to interfacial tension changes. Similarly, anisotropic particles could introduce directional mechanical properties, enabling new suspension behaviors.

#### Applications and Future Directions

The unique properties of water-continuous capillary suspensions make them highly attractive for numerous applications. In biomedicine, the biocompatibility of PEG and dextran makes this system suitable for creating injectable gels with active ingredient encapsulation. Such gels could combine yield stress for structural stability with shear-thinning behavior for ease of injection. In the food industry, this system offers a sustainable alternative to oil-based formulations, allowing for the design of low-calorie products using natural biopolymers. For cosmetics, the ability to tune mechanical properties with small amounts of secondary phase could enable stable emulsions with specific textural properties.

Another promising avenue is the structuring of living systems, such as suspensions containing soft particles like cells. The ability to finely control the mechanical environment of these suspensions may be critical for biomedical applications, such as cell scaffolds or delivery systems. Additionally, environmentally friendly applications, such as water-borne paints, stand to benefit from the elimination of oil-based components.

In summary, this work lays the foundation for further exploration of water-continuous capillary suspensions, highlighting their versatility and potential for broad scientific and industrial impact. Future studies focusing on the interplay between phase separation, particle properties, and rheology will be essential to fully harness the potential of these systems.

## Conclusions

This study demonstrates that the phase-separating system of PEG and dextran in water (ATPS) can be used to tune the rheology of water-continuous suspensions through the introduction of capillary forces. Using cloud-point titration, we successfully characterized the PEG-dextran ATPS and produced two distinct aqueous phases as liquid phases for capillary suspensions.

A robust protocol was developed for creating water-continuous, oil-free capillary suspensions. Particles suspended in the dextran-rich phase and a small amount of the PEG-rich phase added led to the formation of capillary bridges. This resulted in a visible transition in flow behavior from liquid-like to gel-like, confirmed by confocal microscopy and oscillatory rheology. The addition of just 1% PEG-rich phase induced a yield stress of 8–10 Pa and increased the storage modulus by 5 orders of magnitude. The rheological properties were tunable, with the storage modulus maximized at 0.5–1% PEG-rich phase, beyond which particle-filled droplets weakened the gel structure.

Frequency sweep measurements revealed a Maxwell-like response at low frequencies, governed by the capillary gel’s relaxation time, and a high-frequency response dominated by the viscous contribution of the dextran matrix. These findings emphasize the significant impact of capillary forces on the viscoelastic properties of aqueous capillary suspensions and demonstrate their versatility as a tool for tailoring suspension rheology. The approach presented here provides a foundation for further studies and offers valuable insights for scientific and industrial applications.

## Supporting Information Available

Images illustrating cloud-point titration samples of PEG–dextran aqueous two-phase systems (Figures S1 and S2), and data and analysis on dilution lines for phase behavior and critical point determination (Figure S3), and microscopic images of silica particles (Figure S4) (PDF)
