# Supplementary material for: Tunable Capillary Suspensions from Aqueous Two-Phase Systems
Source: Langmuir. 2025 Apr 28;41(18):11604–13. doi: 10.1021/acs.langmuir.5c00749 (PMC12080324; doi:10.1021/acs.langmuir.5c00749)
Supplement: Supplementary file 1 — la5c00749_si_001.pdf [file la5c00749_si_001.pdf]

Supporting Information: Tunable capillary suspensions from aqueous two-phase systems

Leonardo Ruiz-Martínez,[†] Frans Leermakers,[†] Simeon Stoyanov,[†,‡] and Jasper van der Gucht[*,†]

†*Physical Chemistry and Soft Matter, Wageningen University and Research, Wageningen 6708 WE, the Netherlands*

‡*Food, Chemical, and Biotechnology cluster, Singapore Institute of Technology, 10 Dover Drive, Singapore, Singapore, 138683 Singapore*

E-mail: jasper.vandergucht@wur.nl

Contents

Cloud-point titration: Visual demonstration **S-2**

Dilution lines and critical point determination **S-4**

Microscopic imaging of silica particles **S-5**

Cloud-point titration: Visual demonstration

The following images illustrate the phase behavior of the PEG-dextran aqueous two-phase system (ATPS) during cloud-point titration. Figure S1 shows a clear single-phase solution, while Figure S2 shows a cloudy solution at the onset of phase separation. These transitions are used to determine the critical point for phase coexistence.

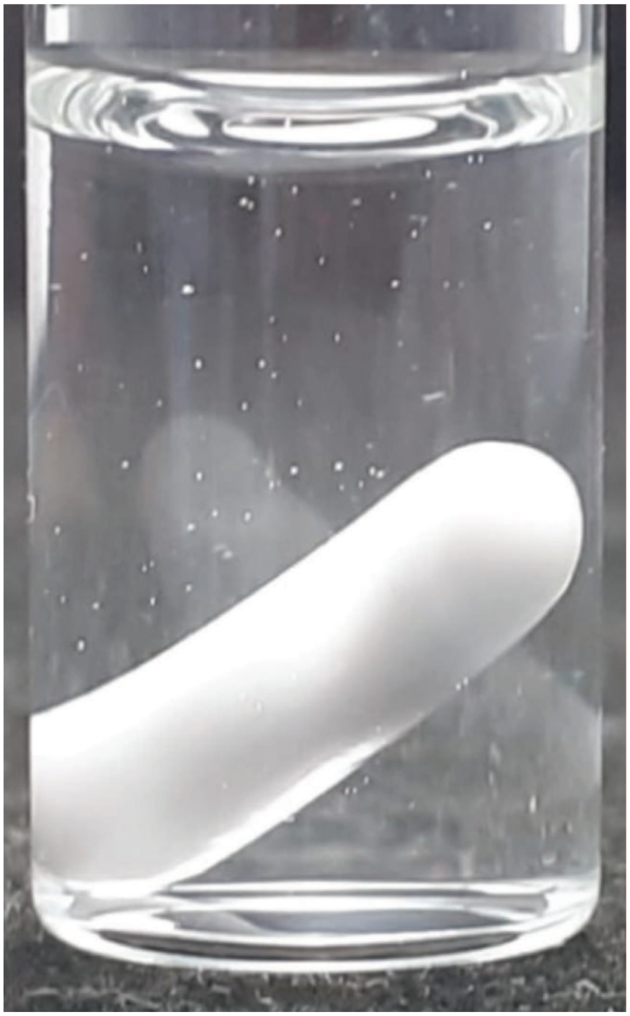

Figure S1: Clear single-phase solution during cloud-point titration.

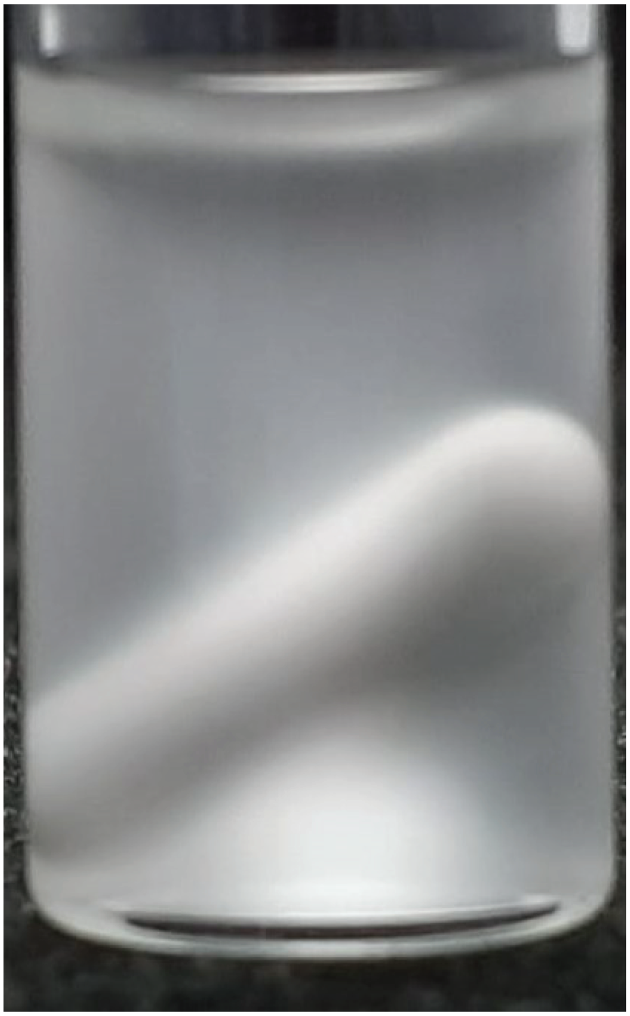

Figure S2: Cloudy two-phase solution indicating the onset of phase separation during cloud-point titration.

Dilution lines and critical point determination

The phase behavior of the PEG-dextran ATPS was further analyzed using dilution lines. Figure S3 shows the volume fraction of the dextran-rich phase (Φ^d) as a function of the distance from the binodal ($w/w_{bi} - 1$) for various weight ratios of dextran to PEG (w_d/w_p). The critical point in this study is determined as the point where the dilution line for $w_d/w_p = 4.67$ intersects near $\Phi^d = 0.5$ when approaching the binodal.

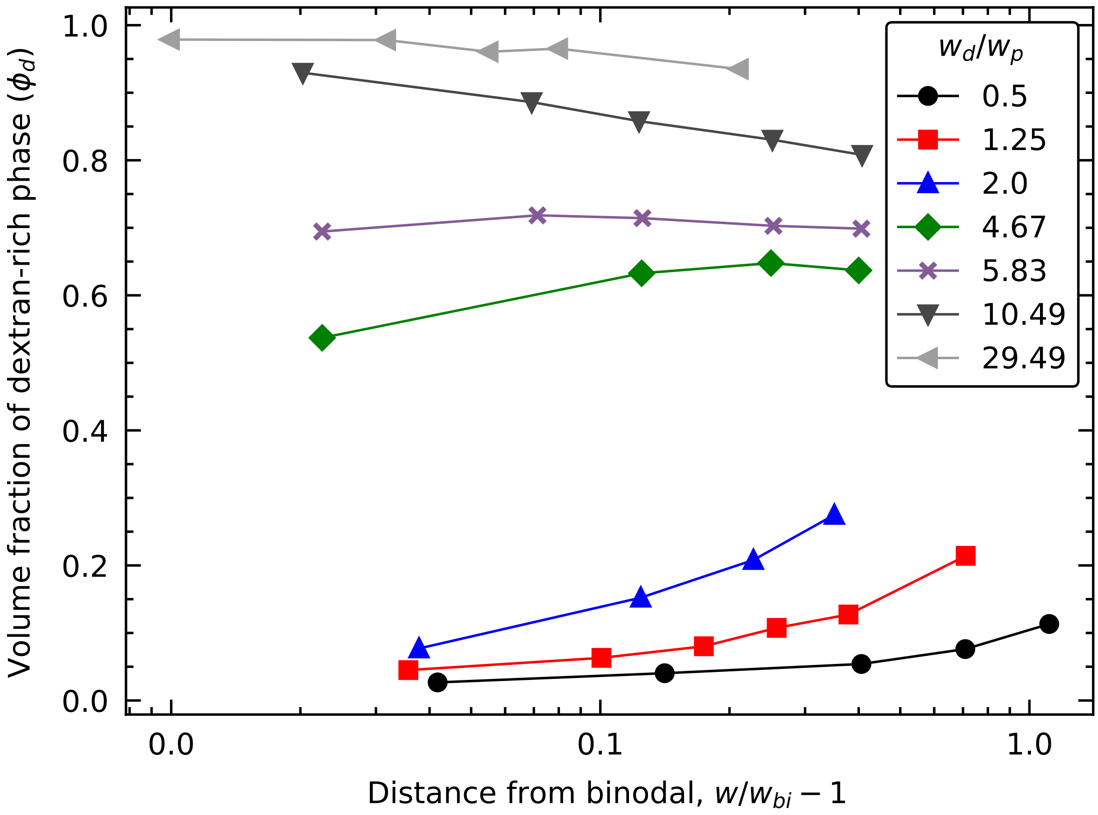

Figure S3: Volume fraction of dextran-rich phase (Φ^d) for different weight ratios (w_d/w_p) along dilution lines.

Microscopic imaging of silica particles

To confirm the morphology of the silica particles used in this study, we acquired a bright-field microscopic image at 50× magnification. The image (Figure S4) clearly shows that the particles are spherical, with no evidence of merging.

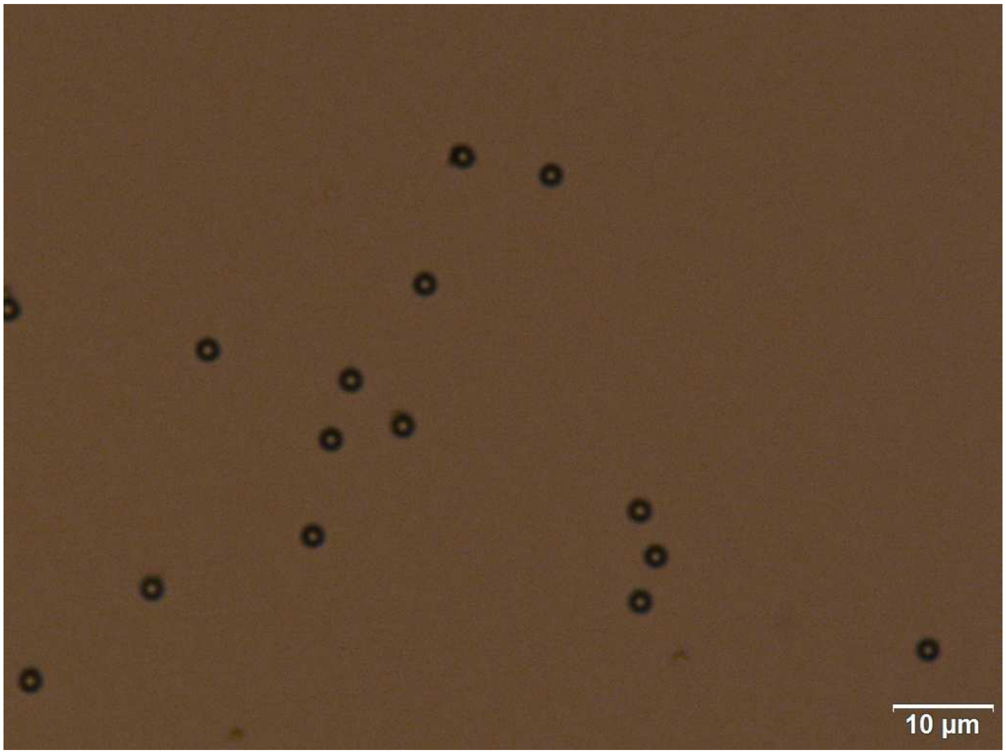

Figure S4: Bright-field microscopic image of silica particles at 50× magnification, confirming their spherical morphology.
